# Predictive Value of Temporal Muscle Thickness Measurements on Cranial Magnetic Resonance Images in the Prognosis of Patients With Primary Glioblastoma

**DOI:** 10.3389/fneur.2020.523292

**Published:** 2020-11-12

**Authors:** Fang Liu, Dong Xing, Yunfei Zha, Li Wang, Wei Dong, Liang Li, Wei Gong, Lei Hu

**Affiliations:** ^1^Department of Radiology, Renmin Hospital of Wuhan University, Wuhan, China; ^2^Renmin Hospital of Wuhan University, Wuhan, China; ^3^Department of Gastroenterology, Renmin Hospital of Wuhan University, Wuhan, China

**Keywords:** glioblastoma, temporal muscle thickness, MRI, overall survival time, sarcopenia

## Abstract

**Objective:** To investigate the predictive value of prognosis of primary GBM patients using TMT on three-dimensional (3D) MR images of the brain.

**Methods:** Data of 130 patients with primary GBM from the TCGA-GBM database were analyzed retrospectively. TMT was measured on the axial plane by multi-planar reformation (MPR) of T1WI MR images perpendicular to the long axis of the temporal muscle at the level of the orbital roof. The axial MR plane was oriented parallel to the anterior commissure-posterior commissure line. Student's *t*-test or Mann–Whitney *U*-test was utilized to determine whether there were significant differences in the TMT and OS between male and female patients. The Pearson correlation analysis was adopted to evaluate the correlation between the age at GBM diagnosis and TMT. All patients were divided into two groups based on their median TMT, and the Kaplan–Meier curve was used to calculate the OS curve. The association between TMT and OS of GBM patients, as well as the multivariate analysis of TMT and other clinical factors affecting the survival time, was evaluated with Cox regression model.

**Results:** TMT was a risk factor for the prognosis of GBM with its hazard ratio (HR) of 0.802 (95% CI 0.698–0.922; *P* = 0.002; Cox regression model). Grouped by median TMT, the group with above-median TMT demonstrated a significant increase in survival time (15.6 months) compared with the one with below-median TMT (11.2 months) (*P* < 0.001; log-rank test). In the multivariate survival analysis using a Cox regression model, TMT (HR 0.863; 95% CI 0.748–0.996; *P* = 0.044), age at the diagnosis of GBM (HR 1.042; 95% CI 1.024–1.060; *P* < 0.001), and concurrent chemoradiotherapy (HR 0.510; 95% CI 0.336–0.775; *P* = 0.002) were significantly associated with survival time.

**Conclusion:** TMT as an independent predictor is sensitive to the survival prognosis of primary GBM patients, which has potential to predict the survival time.

## Introduction

Glioblastoma (GBM) is the most universal and invasive primary malignant intracranial neoplasm, WHO grade IV, accounting for ~50% of the population of gliomas (1, 2). Patients with such a high-grade malignancy have poor prognosis despite intricate treatments, with the median survival of about 14.6 months and the 5-year survival rate of <10% (2, 3). At present, the full Stupp protocol is universally recognized as a standard treatment for newly diagnosed GBM patients since 2005, which is surgical resection to the practically possible extent and then followed by radiotherapy combination with temozolomide (TMZ). Given the heterogeneity of GBM, the optimal treatment strategy should target individualized therapies. Therefore, the first imperative is to confirm clinical indicators that can effectively predict the prognosis of GBM. Some current studies have pointed to indicators, such as age, tumor location, WHO grade, KPS score, degree of resection, postoperative concurrent chemoradiotherapy, and regulatory genes, for the prognosis of GBM (4, 5).

Recent studies have found that the survival time of patients with brain metastases and recurrent GBM is closely related to temporal muscle thickness (TMT) (6–8). However, no researches have to date confirmed the correlation between the prognosis of primary GBM patients and TMT.

This study aimed to determine the predictive value of TMT in the prognosis of primary GBM patients, which will shed light on further individualized treatment.

## Materials and Methods

### Clinical Data

The clinical and imaging data of all GBM patients were derived from the Cancer Genome Atlas Glioblastoma Multiforme (TCGA-GBM) of The Cancer Imaging Archive (TCIA) (9) (https://wiki.cancerimagingarchive.net/display/Public/TCGA-GBM).

The inclusion criteria were as follows: (i) patients pathologically diagnosed as primary (*de novo*) untreated GBM and (ii) available preoperative imaging data of 3D cranial MR plain or contrast-enhanced images with good quality. The exclusion criteria encompassed (i) incomplete clinical data; (ii) patients aged <18 years; (iii) cranial MR images with poor quality; and (iv) cranial surgery infringing the temple. Overall survival (OS) was defined as the period from the diagnosis of GBM to death. If the patient was still alive at the last visit, the data would be recorded as right censored. One hundred thirty GBM patients (81 males and 49 females) were included in the study cohort, with the age ranging from 18 to 86 years. The average age at the diagnosis of primary GBM was 60.02 ± 14.33 years (mean ± standard deviation). An overview of patient characteristics and clinical data is shown in Table 1.

**Table 1:** Demographic characteristics and clinical data of 130 primary glioblastoma (GBM) patients.

**Characteristics of GBM patients**	***n***	**%**
Median age at the diagnosis of GBM, years (range)	61.5 (18–86)	
Median overall survival from diagnosis of GBM, months (range)	10.2 (0.17–66.23)	
Overall survival of male patients, months (*mean ± SD*)	12.76 ± 11.34	
Overall survival of female patients, months (*mean ± SD*)	14.11 ± 12.35	
Gender		
Male	81	62.31
Female	49	37.69
Treatment		
Concurrent chemoradiotherapy	95	73.08
Non-concurrent chemoradiotherapy	35	26.92
Alive at last follow-up		
Yes	24	18.46
No	106	81.54

For all imaging and clinical data were publicly available, the study was exempt from the approval of the Institutional Review Board.

### Measurement of Temporal Muscle Thickness on Cranial MRI

**Figure 1:**
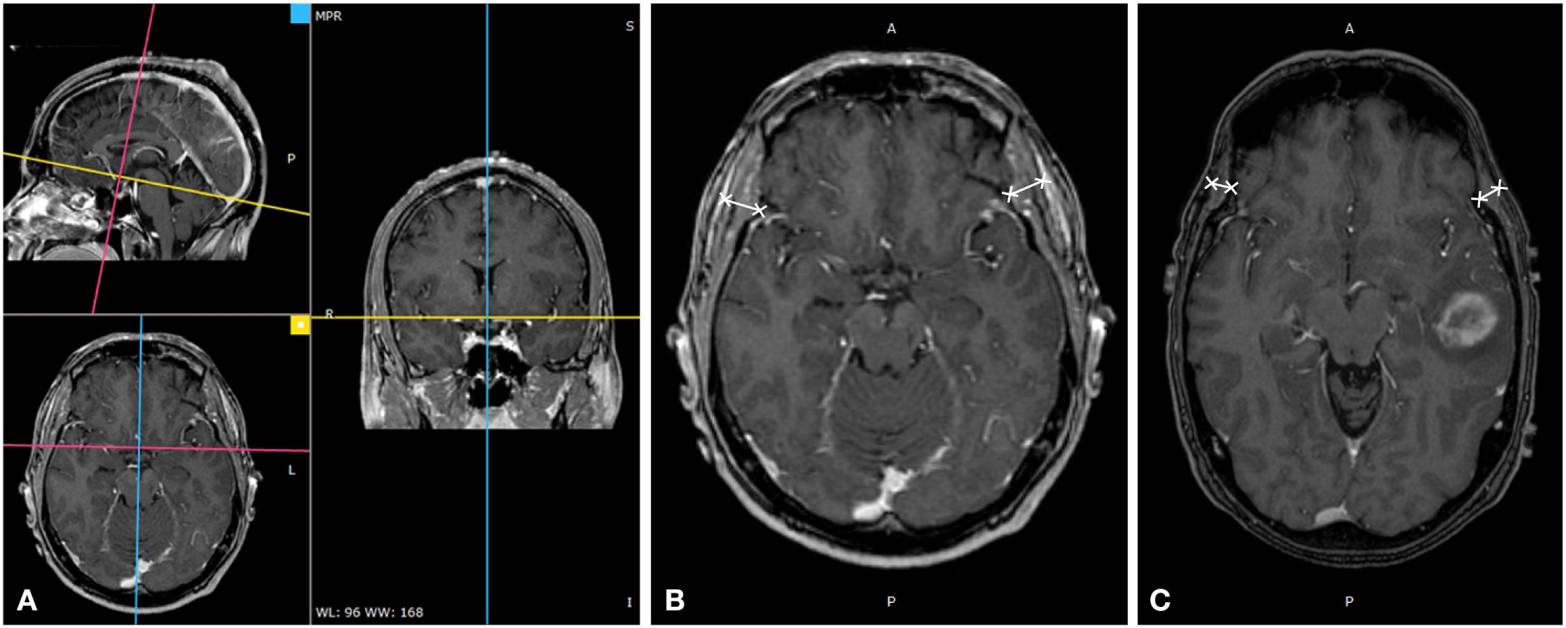
Temporal muscle thickness (TMT) measurement on cranial MRI. **(A)** TMT measurement on cranial thin T1WI contrast-enhanced image through multiplanar reformation, **(B)** A 49-year-old male patient with an overall survival of 38.1 months (bilateral mean TMT = 10.20 mm), and **(C)** a 57-year-old male patient with an overall survival of 16.9 months (bilateral mean TMT = 5.94 mm).

All 3D cranial T1WI MR plain radiographs or contrast-enhanced images (slice thickness 0.9–1.5 mm), in DICOM format, were imported into an offline workstation, and TMT was manually measured using the RadiAnt DICOM Viewer software (Version 4.6.9). The process of the measurement was described as follows (6). When reading the eligible multi-planar reformation (MPR) of T1WI MR plain radiographs or contrast-enhanced images on the axial plane, an experienced radiologist measured the left and right baseline TMT perpendicular to the long axis of the temporal muscle at the level of the orbital roof. The axial MR imaging was oriented parallel to the anterior commissure-posterior commissure line. Moreover, the Sylvian fissure was defined as a referenced anatomical landmark regarding the anterior–posterior direction. To enhance the accuracy of each assessment, we determined these landmarks before TMT measurements. TMT of the left and right side was averaged to obtain the mean value that was taken as the TMT of each patient, as shown in Figure 1.

TMT analyses were performed independently by a radiologist with 5-year experience in MR diagnosis. Another radiologist with a 4-year experience in MR diagnosis conducted repetitive measurements of TMT in randomly selected 30 patients. Clinical features and survival data of the patients were blind to the radiologists during the TMT measurement.

### Statistical Analysis

Statistical analysis was performed using SPSS version 19.0 (IBM Corporation, Armonk, NY, USA). The intraclass correlation coefficient (ICC) was used to assess the reliability of two observers. Student's *t*-test or Mann–Whitney *U*-test was utilized to determine whether there were significant differences in the TMT and OS between male and female patients. The Pearson correlation analysis was adopted to evaluate the correlation between the age at GBM diagnosis and TMT. All patients were divided into two groups based on their median TMT. The Kaplan–Meier curve was used to calculate the OS curve, and the log-rank test was applied to investigate differences in OS between the two groups. The association between TMT and OS of GBM patients, as well as the multivariate analysis of TMT and other clinical factors affecting the survival time, was evaluated with the Cox regression model. A *P*-value of <0.05 was considered statistically significant.

## Results

### Assessment of TMT and Its Correlation With Clinical Characteristics

The mean of right TMT of the patients was 9.31 ± 1.62 mm (ranging from 4.72 to 14.90 mm), the mean of left TMT was 9.25 ± 1.66 mm (ranging from 4.28 to 13.88 mm), and the mean TMT was 9.31 ± 1.62 mm (ranging from 4.50 to 14.39 mm). The ICC of left- and right-sided TMT calculated by two radiologists was 0.878 and 0.895 (*P* < 0.001). The mean TMT in males was 9.59 ± 1.60 mm, significantly greater than that of 8.83 ± 1.54 mm in females, with a statistically significant difference (*t* = −2.662, *P* = 0.009). There was no significant difference in the OS between males (12.76 ± 11.34 months) and females (14.11 ± 12.35 months) (*Z* = −0.401, *P* = 0.688). Pearson correlation analysis showed a slight correlation between mean TMT and the age at the diagnosis of GBM (*r* = −0.173, *P* = 0.049).

### Analysis for the Efficacy of TMT in Predicting the Prognosis of Patients With GBM

**Figure 2:**
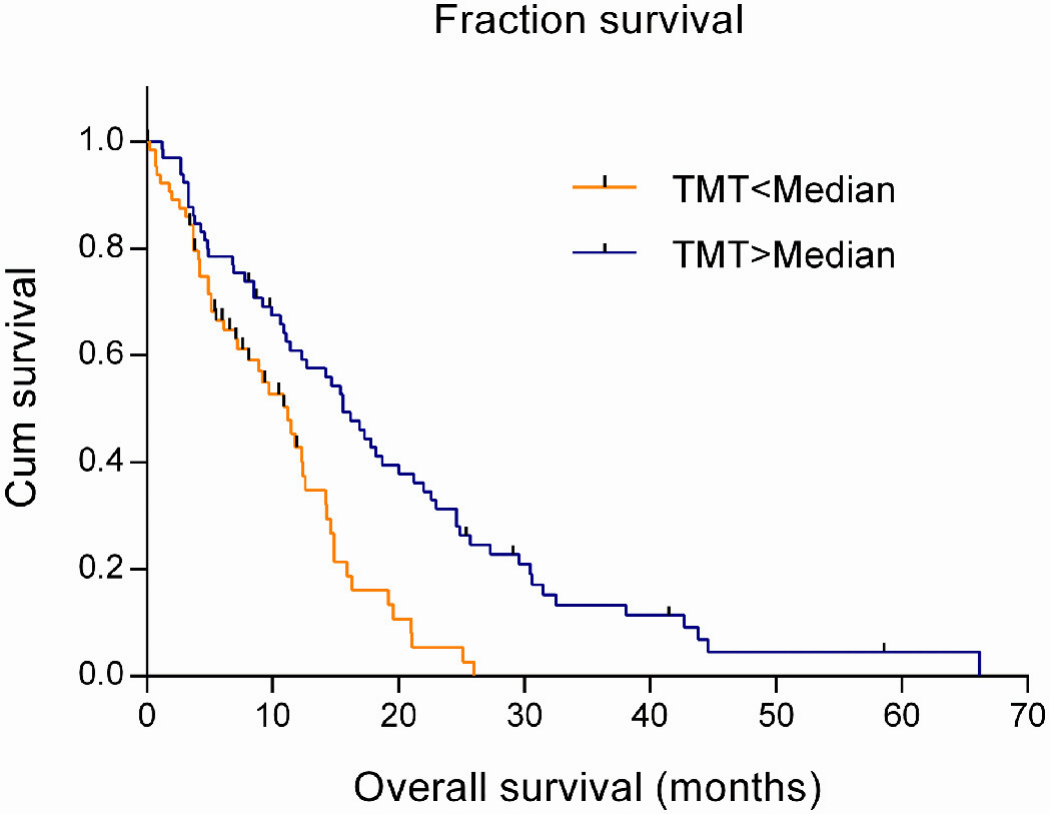
Overall survival according to median TMT of 130 primary GBM patients.

The estimation in a Cox regression model showed that TMT was a risk factor related to the prognosis of GBM patients, with the hazard ratio (HR) of 0.802 (95% CI 0.698–0.922; *P* = 0.002). That meant the risk of death would increase by 19.8% with every one-millimeter decrease in TMT. Using the Kaplan–Meier model and the log-rank test, it was found that the survival time significantly prolonged in patients with above-median TMT (median, 15.6 months) compared with those with below-median TMT (median, 11.2 months) (χ^2^ = 13.665, *P* < 0.001), as shown in Figure 2.

### Multivariate Analysis of Factors Affecting the Survival of GBM Patients

In the multivariate survival analysis using a Cox regression model, TMT (HR 0.863; 95% CI 0.748–0.996; *P* = 0.044), age at the diagnosis of GBM (HR 1.042; 95% CI 1.024–1.060; *P* < 0.001), and concurrent chemoradiotherapy (HR 0.510; 95% CI 0.336–0.775; *P* = 0.002) were significantly associated with the survival time of GBM patients. There was no significant association between the survival of GBM patients and gender (*P* = 0.063).

## Discussion

This study aimed at exploring the correlation between TMT and the survival time in patients with primary GBM. The results suggested that TMT was an independent risk factor for primary GBM patients, which might be a contributor to improve the prognosis prediction of this malady.

At present, a growing number of researches have drawn solicitous attention to the correlation between TMT and the survival time of patients with brain tumors. Furtner et al. (6, 7) found that the TMT measured on MRI was closely related to the survival of patients with brain metastases from breast cancer, lung cancer, and melanoma, with the mortality risks decreasing by 19, 24, and 27.9%, respectively, with every one-millimeter increase in TMT. Patients with above-median TMT presented significantly longer OS than those with below-median TMT. In our study, the mortality risk in primary GBM patients decreased by 19.8% with every one-millimeter increase in TMT. Moreover, patients with above-median TMT showed the median survival time of 15.6 months, much longer than 11.2 months in those with below-median TMT, which was consistent with the published literature. Recently, Furtner et al. (8) also found that TMT was closely related to the OS and progression-free survival of the patients with recurrent GBM. However, Furtner's study could not rule out the direct or indirect effect of glioma resection on TMT as the GBM subjects were postoperative patients. As the patients diagnosed with GBM were preoperative in our study, the influence factors from the brain surgery could be excluded. Then, the independent influence of TMT on the OS of GBM patients could be better verified.

Not only in GBM, but also in a variety of diseases, low skeletal muscle mass was increasingly recognized as a biomarker of unfavorable prognosis (10). Some studies believed that the skeletal muscle mass of tumor patients closely associates with their quality of life, therapeutic efficacy, tumor recurrence, and so on (11). Diseases such as cachexia or sarcopenia that seriously affected the survival of tumor patients were mainly characterized by reduced skeletal muscle mass and function (12). Therefore, the accurate assessment of skeletal muscle mass had critical clinical implications.

The third lumbar vertebra skeletal muscle index (L3-SMI) was one of the most widely used methods for evaluating skeletal muscle mass. L3-SMI was used to calculate the skeletal muscle cross-sectional area (CSA) at the L3 vertebral level on CT or MRI by thresholding, and then the skeletal muscle area (SMA) was divided by the square of the patient's height [SMI = SMA/height^2^ (cm^2^/m^2^)] (13). Several studies showed that L3-SMI, which could be calculated based on routine abdominal CT or MRI examinations, had a good correlation with the skeletal muscle mass. As these methods were more convenient and efficient than dual-energy X-ray absorptiometry (DXA), bioelectrical resistance measurement (BIA), and other skeletal muscle measurements, L3-SMI had become an objective and effective index for evaluating muscle mass in clinical practices (10, 11).

However, a considerable number of patients with brain tumors did not routinely undergo abdominal CT or MR examinations at the L3 level, and additional scans could bring more economic burden and extra radiation exposures to the patients. In recent years, some studies reported that TMT could be used as a substitute for L3-SMI in patients with head and neck cancer. Ranganathan et al. (14) found a correlation between TMT and horizontal psoas muscle area at the L3 level. Leitner et al. (15) reckoned that TMT in brain metastasis patients was closely related to skeletal muscle CSA and SMI at the L3 level. The American Society of Parenteral and Enteral Nutrition reached a consensus that temporal muscle atrophy was one of the physical manifestations of muscle atrophy in malnutrition, which could be used for the nutritional assessment of patients (16). Hasegawa et al. (17) adopted ultrasound to measure TMT, which proved that TMT could be used as a predictor of nutritional status in the elderly. However, the interoperability of ultrasound for TMT measurement was susceptible to the operation of different sonographers and required specialized operational training (17). In this study, the ICC of the left and right TMT was 0.878 and 0.895, respectively, demonstrating a good consistency of TMT measurements on routine cranial MRI. In addition, the measurement of SMA required professional semiautomatic segmentation software (18). Its relatively complicated procedure (15, 19) resulted in a longer learning curve and was time-consuming for a tester. For example, each CSA measurement might take about 25 min. In contrast, TMT measurement was merely superficial and comfortable with excellent reliability of different doctors and needed only 30 s per patient (6, 8, 15). Therefore, we believe that TMT is suitable for daily clinical application.

However, some studies yielded inconsistent results in TMT values. Furtner et al. (6, 7) reported a median TMT of 5.4 mm in the breast cancer brain metastasis population and 5.9 mm in non-small cell lung cancer brain metastasis patients. In melanoma patients with newly diagnosed brain metastases, the overall mean TMT was 5.8 mm, and for the recurrent GBM patients, the median TMT was 7.1 mm. Leitner et al. (15) reported mean TMT in lung cancer and melanoma patients of 6.1 and 6.2 mm, respectively. Donizetti et al. (20) calculated that the mean thickness of anterior temporal muscles in patients with Parkinson's disease and control groups in the maximum voluntary contraction (MVC) was 8.3 and 7.2 mm, respectively. Ranganathan et al. (14) reported the mean TMT in adult trauma patients of 9.0 mm. Steindl et al. (21) found that TMT values in the retrospective healthy cohort ranged from 3.75 to 15.75 mm, and the mean TMT of male volunteers in 40–49 years group reached 10.0 mm. Song et al. (22) performed a cadaveric study and found TMT ranging from 14.0 ± 3.2 mm (anterior) to 5.9 ± 1.6 mm (posterior). In our study, the median TMT in the primary GBM patients was 9.2 mm. We speculated that the disparity in TMT values might attribute to types of underlying diseases, diverse clinical characteristics (such as age and gender), various disease courses of diagnosis, and different measurement procedures among the cohorts.

Our study found that there was a weak correlation between TMT and age at the diagnosis of GBM, which indicated that the age of patients had little effect on TMT, and TMT was mainly related to their nutritional status. Our study also showed that although the mean TMT of male patients was greater than that of female patients, no distinguished difference existed in the OS between male and female patients. It suggested that TMT was adequate for the prognosis prediction of primary GBM, independent of patient gender. These findings were consistent with previous reports (6, 7, 15).

The limitations of our study must be acknowledged. Firstly was the lack of some important information that influenced survival times of GBM patients—for example, molecular data such as MGMT promotor methylation and information on the actual surgical treatment was a major limitation in our study. Secondly, to reduce the partial volume effect, thin-layer cranial MR images of 1 mm × 1 mm × 1 mm were used in the literature. However, the layer thickness in some patients reached 1.5 mm in this study, which might cause some deviations in the TMT measurement. Thirdly, TMT could be affected by dental or oral diseases (7, 23). In our study, we did not obtain the history of dental or oral diseases of the subjects. To eliminate the interference from dental or oral diseases as much as possible, we measured both-sided TMT and calculated the mean TMT for each GBM patient. However, such an effect still existed.

In short, we confirm that the measurement of TMT is a simple, convenient, and repeatable method. As TMT has a significant correlation with the survival time of primary GBM patients, it can be used as an independent prognostic factor of the disease, which may benefit early individualized treatments for the patients.

## Data Availability Statement

All datasets generated for this study are included in the article/supplementary material.

## Ethics Statement

Ethical review and approval was not required for the study on human participants in accordance with the local legislation and institutional requirements. Written informed consent for participation was not required for this study in accordance with the national legislation and the institutional requirements.

## Author Contributions

FL, DX, and YZ: conception, design of the research, and drafting the manuscript. FL, LL, WG, and LH: acquisition of the data. DX and LL: analysis and interpretation of the data. YZ, LW, and WD: critical revision of the manuscript. FL, DX, YZ, LW, WD, LL, WG, and LH: final approval of the version to be published. All authors contributed to the article and approved the submitted version.

## Conflict of Interest

The authors declare that the research was conducted in the absence of any commercial or financial relationships that could be construed as a potential conflict of interest.

## Abbreviations

GBM: Glioblastoma
TMT: Temporal muscle thickness
MRI: Magnetic resonance imaging
ICC: Intraclass correlations
OS: Overall survival.
